# Supplementary material for: A conserved cell division protein directly regulates FtsZ dynamics in filamentous and unicellular actinobacteria
Source: eLife. 2021 Mar 17;10:e63387. doi: 10.7554/eLife.63387 (PMC7968930; doi:10.7554/eLife.63387)
Supplement: Figure 5—source data 1. [file elife-63387-fig5-data1.pptx]

## Slide 1
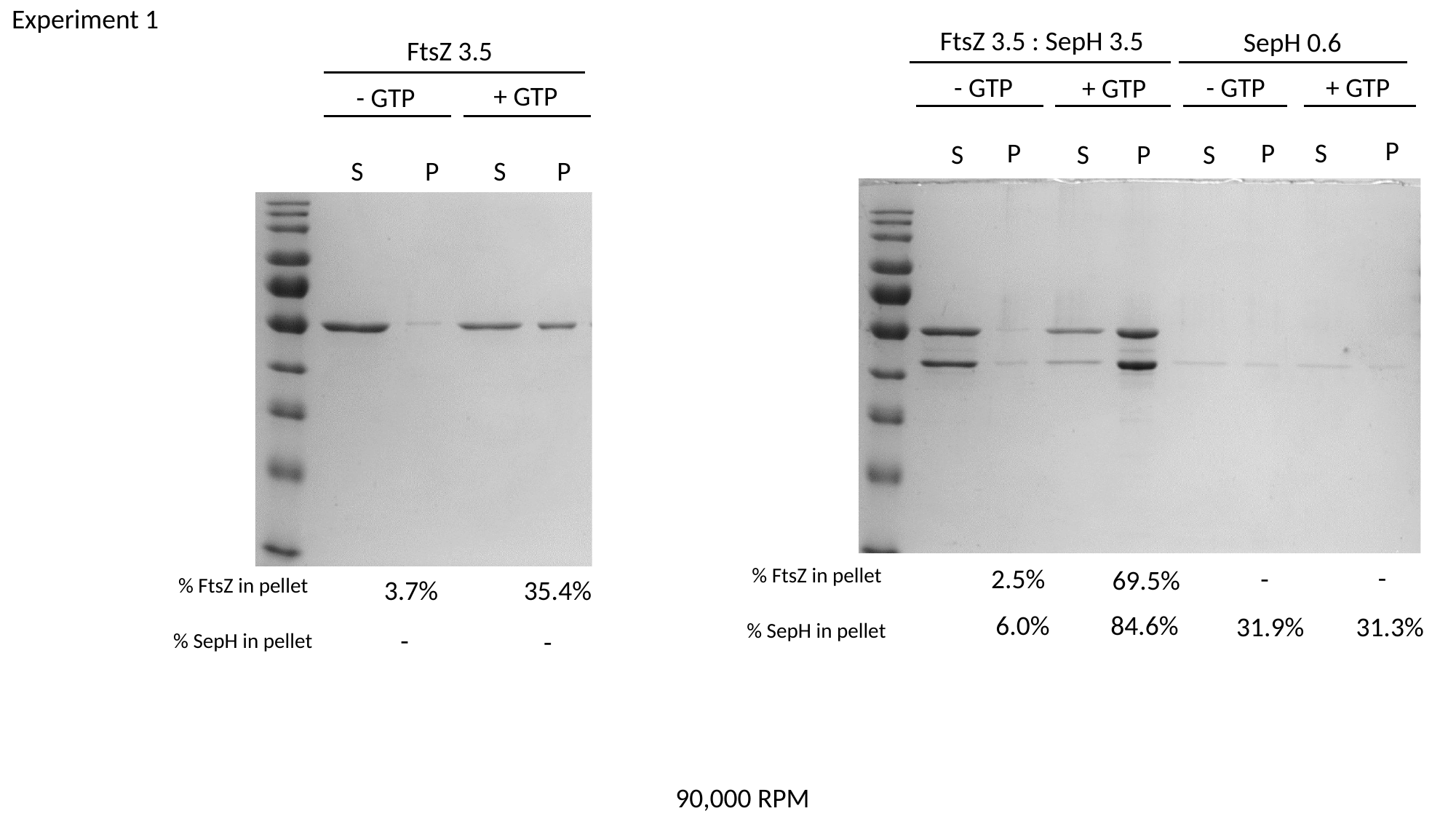

Experiment 1
FtsZ 3.5 : SepH 3.5
SepH 0.6
FtsZ 3.5
- GTP
- GTP
+ GTP
+ GTP
+ GTP
- GTP
P
P
P
S
S
S
P
S
S
P
S
P
-
2.5%
-
% FtsZ in pellet
69.5%
% FtsZ in pellet
3.7%
35.4%
6.0%
84.6%
31.9%
31.3%
% SepH in pellet
-
-
% SepH in pellet
90,000 RPM

## Slide 2
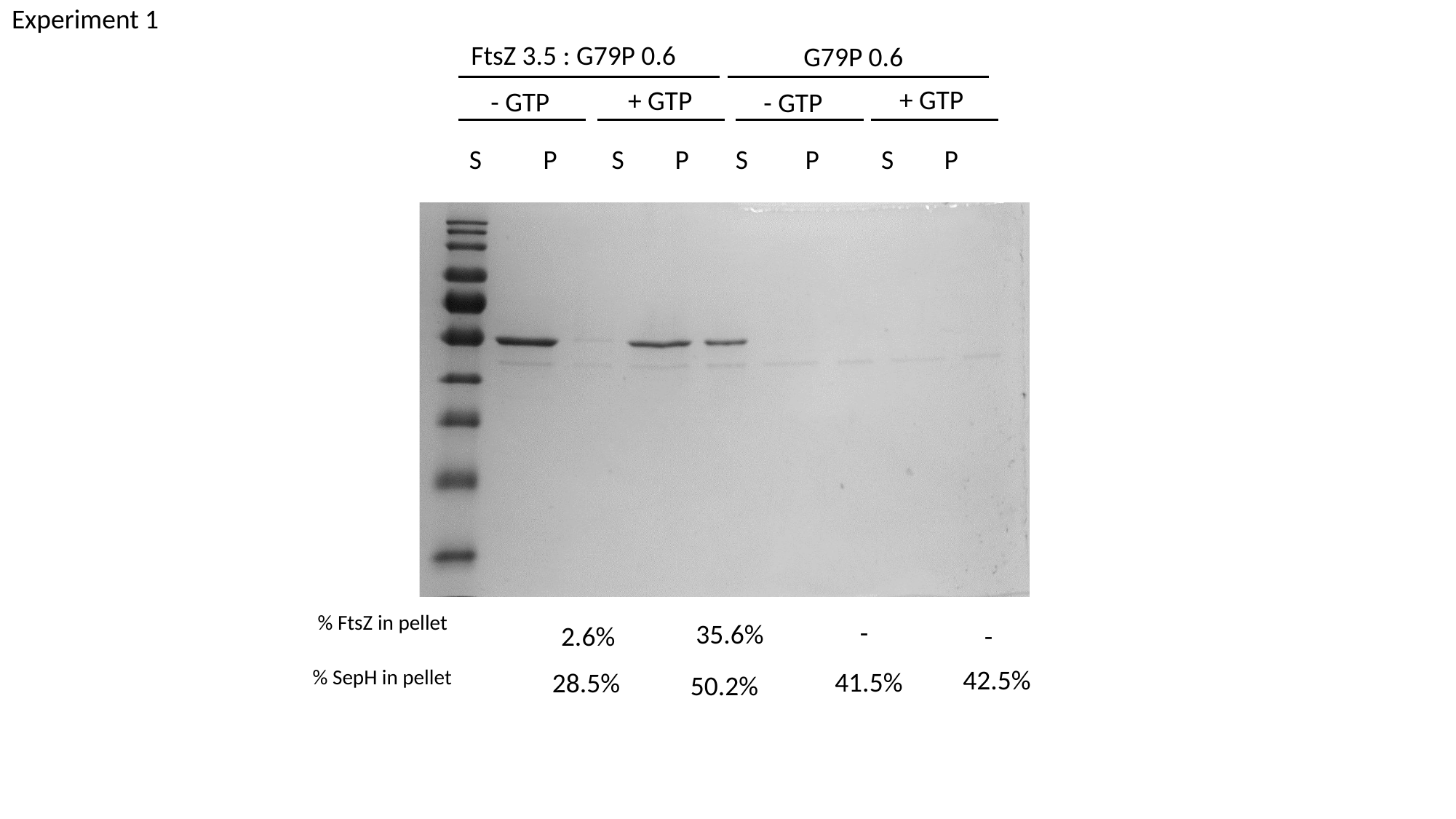

Experiment 1
FtsZ 3.5 : G79P 0.6
G79P 0.6
+ GTP
+ GTP
- GTP
- GTP
S
P
S
P
S
P
S
P
% FtsZ in pellet
-
35.6%
-
2.6%
42.5%
% SepH in pellet
41.5%
28.5%
50.2%

## Slide 3
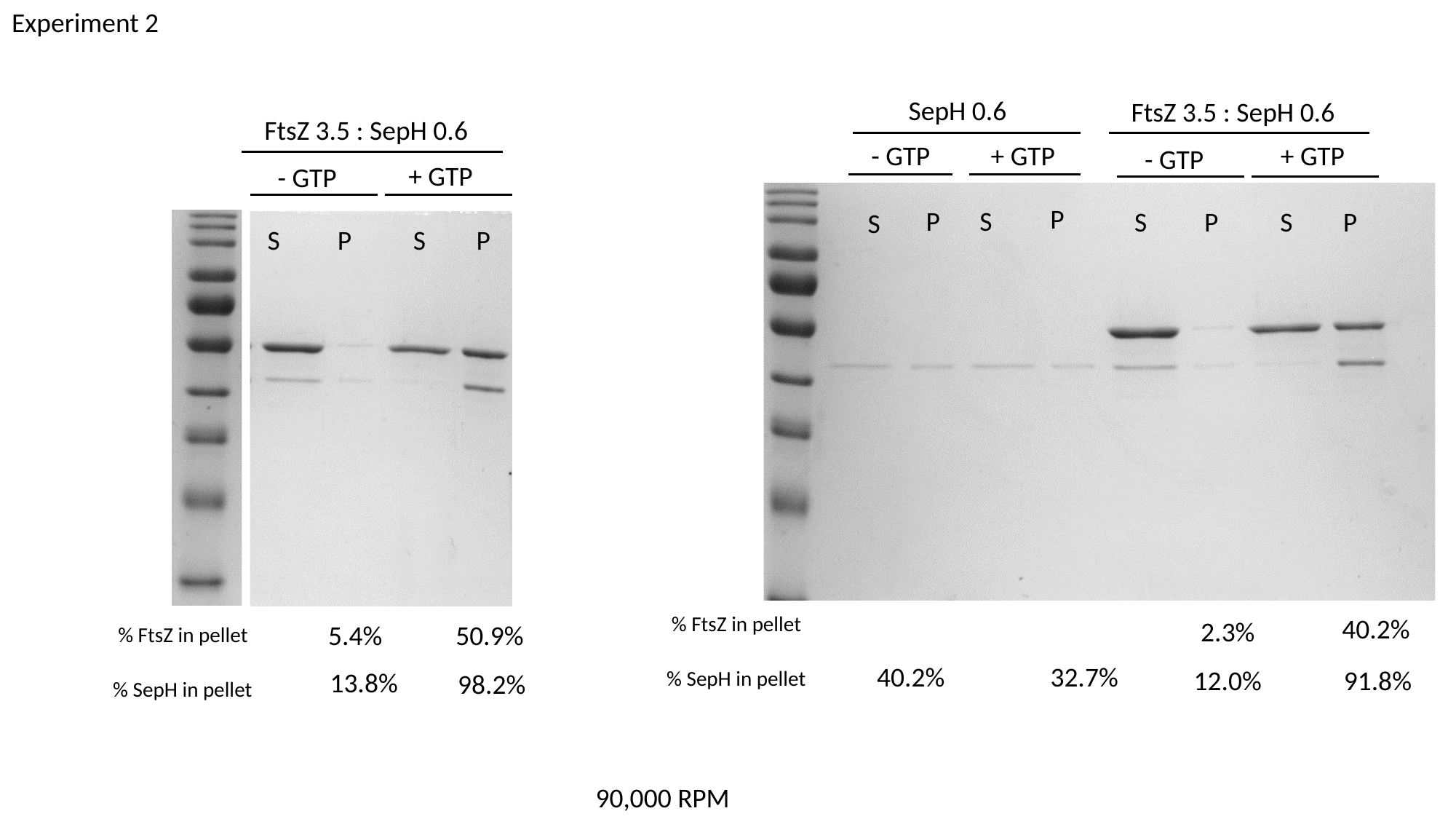

Experiment 2
SepH 0.6
FtsZ 3.5 : SepH 0.6
FtsZ 3.5 : SepH 0.6
- GTP
+ GTP
+ GTP
- GTP
+ GTP
- GTP
P
P
S
S
P
S
P
S
S
P
S
P
% FtsZ in pellet
40.2%
2.3%
5.4%
50.9%
% FtsZ in pellet
40.2%
32.7%
12.0%
91.8%
13.8%
% SepH in pellet
98.2%
% SepH in pellet
90,000 RPM

## Slide 4
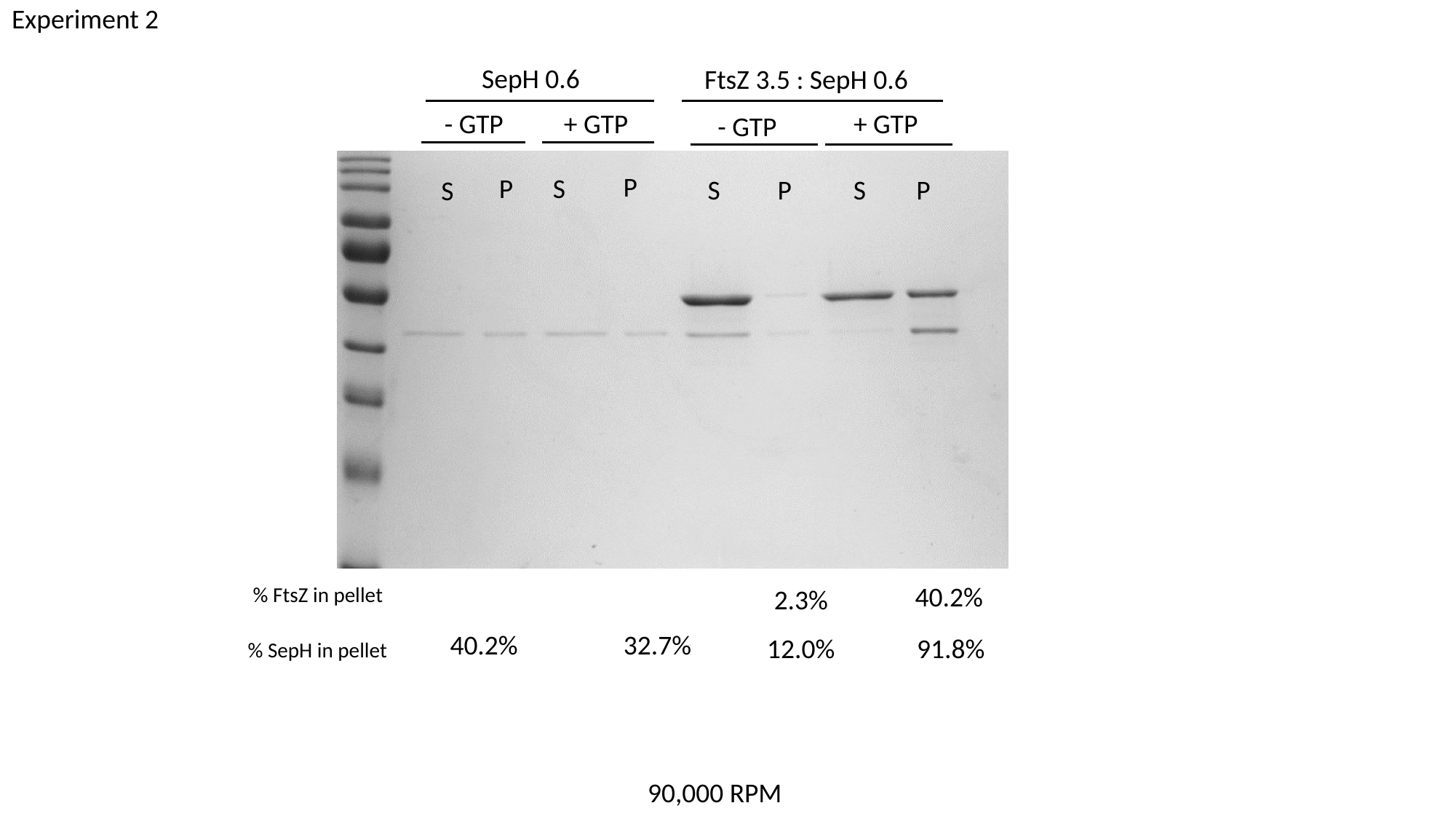

Experiment 2
SepH 0.6
FtsZ 3.5 : SepH 0.6
- GTP
+ GTP
+ GTP
- GTP
P
P
S
S
P
S
P
S
40.2%
% FtsZ in pellet
2.3%
40.2%
32.7%
12.0%
91.8%
% SepH in pellet
90,000 RPM
