# Supplementary material for: A conserved cell division protein directly regulates FtsZ dynamics in filamentous and unicellular actinobacteria
Source: eLife. 2021 Mar 17;10:e63387. doi: 10.7554/eLife.63387 (PMC7968930; doi:10.7554/eLife.63387)
Supplement: Figure 5—figure supplement 4—source data 1. [file elife-63387-fig5-figsupp4-data1.pptx]

## Slide 1
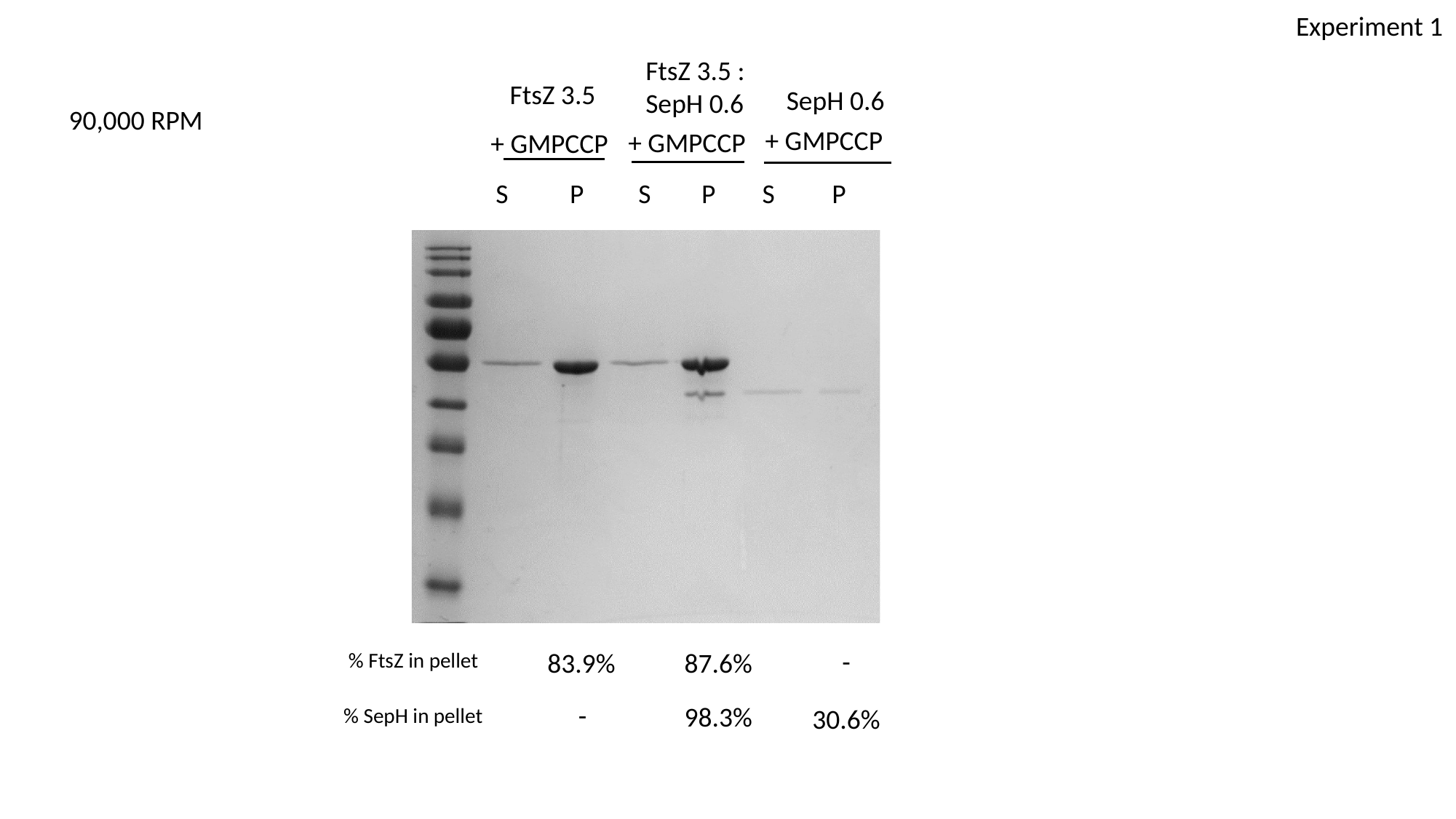

Experiment 1
FtsZ 3.5 : SepH 0.6
FtsZ 3.5
SepH 0.6
90,000 RPM
+ GMPCCP
+ GMPCCP
+ GMPCCP
S
P
S
P
S
P
-
83.9%
87.6%
% FtsZ in pellet
-
98.3%
% SepH in pellet
30.6%

## Slide 2
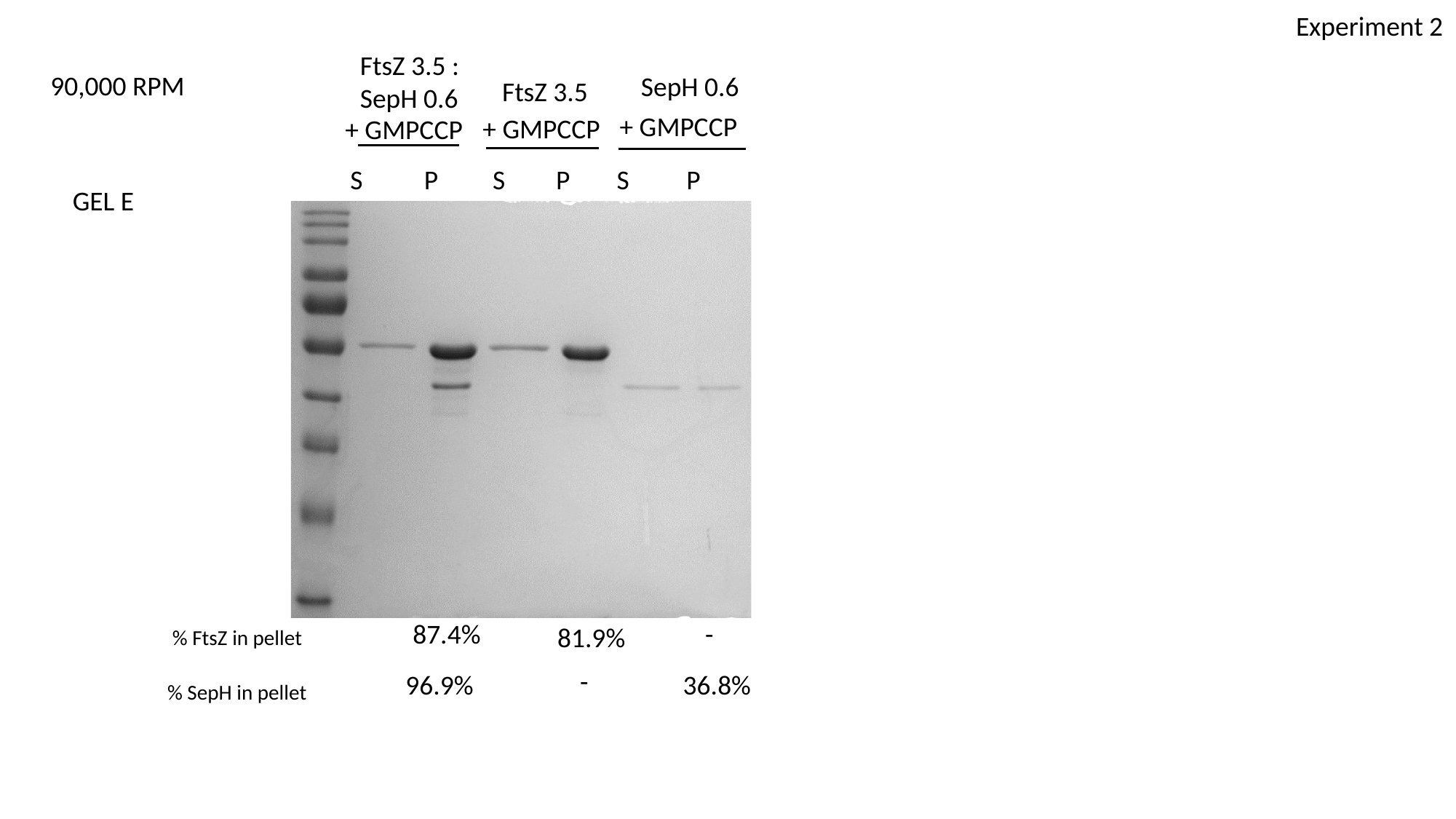

Experiment 2
FtsZ 3.5 : SepH 0.6
90,000 RPM
SepH 0.6
FtsZ 3.5
+ GMPCCP
+ GMPCCP
+ GMPCCP
S
P
S
P
S
P
GEL E
87.4%
-
81.9%
% FtsZ in pellet
-
96.9%
36.8%
% SepH in pellet
