# Supplementary material for: A conserved cell division protein directly regulates FtsZ dynamics in filamentous and unicellular actinobacteria
Source: eLife. 2021 Mar 17;10:e63387. doi: 10.7554/eLife.63387 (PMC7968930; doi:10.7554/eLife.63387)
Supplement: Figure 6—figure supplement 1—source data 1. [file elife-63387-fig6-figsupp1-data1.pptx]

## Slide 1
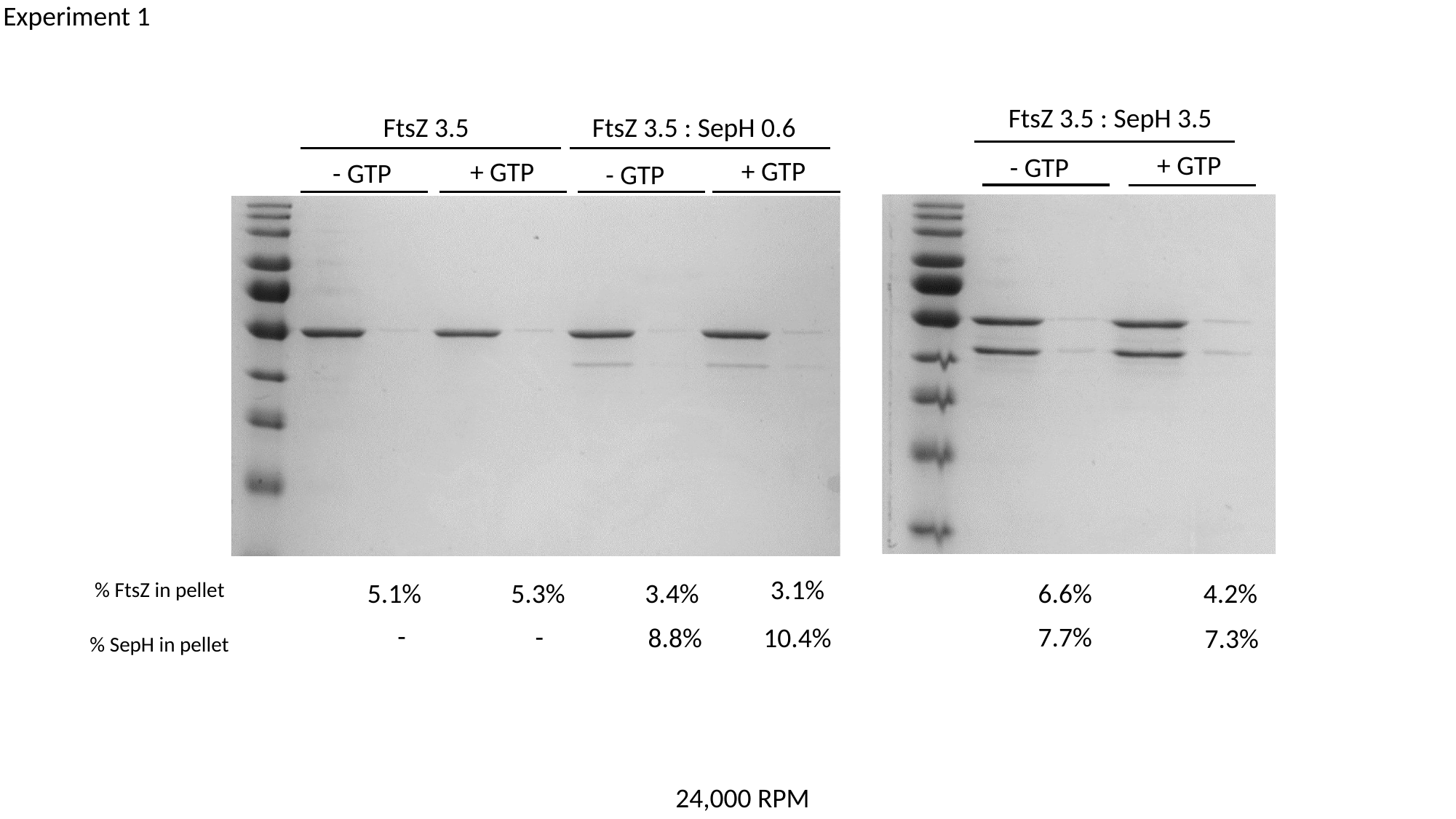

Experiment 1
FtsZ 3.5 : SepH 3.5
FtsZ 3.5
FtsZ 3.5 : SepH 0.6
+ GTP
- GTP
+ GTP
+ GTP
- GTP
- GTP
3.1%
% FtsZ in pellet
5.1%
5.3%
3.4%
6.6%
4.2%
-
-
7.7%
8.8%
10.4%
7.3%
% SepH in pellet
24,000 RPM

## Slide 2
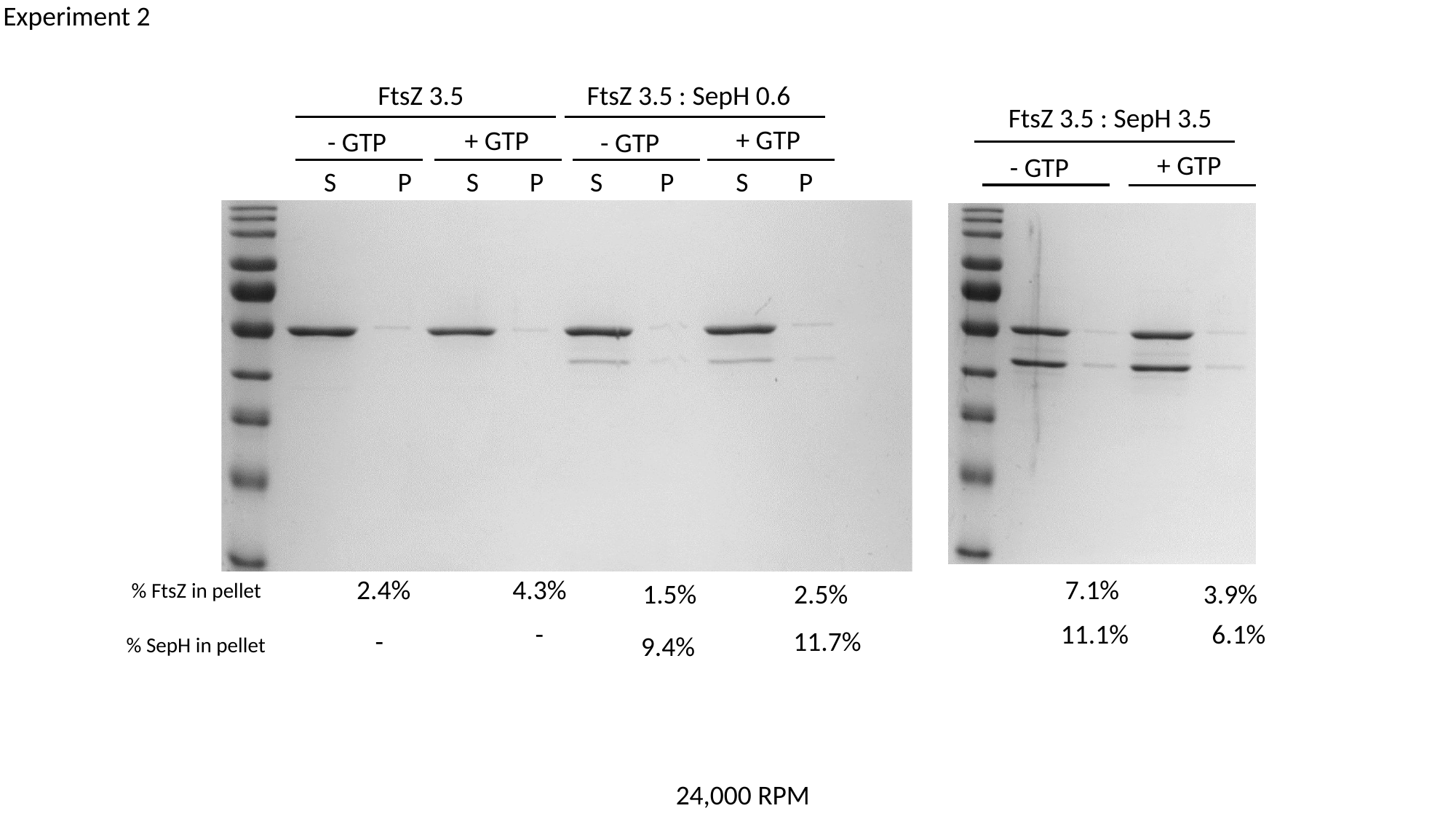

Experiment 2
FtsZ 3.5
FtsZ 3.5 : SepH 0.6
FtsZ 3.5 : SepH 3.5
+ GTP
+ GTP
- GTP
- GTP
+ GTP
- GTP
S
P
S
P
S
P
S
P
2.4%
4.3%
7.1%
1.5%
2.5%
3.9%
% FtsZ in pellet
-
11.1%
6.1%
-
11.7%
9.4%
% SepH in pellet
24,000 RPM
