# Supplementary material for: A conserved cell division protein directly regulates FtsZ dynamics in filamentous and unicellular actinobacteria
Source: eLife. 2021 Mar 17;10:e63387. doi: 10.7554/eLife.63387 (PMC7968930; doi:10.7554/eLife.63387)
Supplement: Supplementary file 1. [file elife-63387-supp1.docx]

**Supplementary File 1**

**Table 1:** Bacterial strains used in this study.

| **Strain** | **Details** | **Source** | **Reference** |
| --- | --- | --- | --- |
| *S.venezuelae* | | | |
| NRRL B-65442 | Wild Type (WT) | Laboratory Strain | NZ_CP018074.1 |
| DU669 | Δ*ftsZ::apr* |  | Santos-Beneit et al., 2017 |
| SS4 | WT *attB_ΦBT1_*_::_pIIJ10257 | pIJ10257 integrated into the ΦBT1-attachment site of *S. venezuelae* | Schlimpert et al., 2017 |
| SS12 | WT *attB*_ΦBT1_::P*_ftsZ_-ftsZ-ypet* | pSS5 integrated into the ΦBT1-attachment site of *S. venezuelae* | Schlimpert et al., 2017 |
| SV56 | Δ*sepH*::*apr* | Chromosomal *sepH* locus was replaced by *apr-oriT* cassette amplified with primer mb118/mb119 and then transduced into WT using ΦSV1 | This work |
| MB747 | Δ*sepH::apr* *attB_ΦBT1_*::P*_sepH_-sepH* | pMB557 integrated into the ΦBT1-attachment site of SV56 | This work |
| MB749 | Δ*sepH::apr* *attB_ΦBT1_*::pIJ10770 | pIJ10770 integrated into the ΦBT1-attachment site of SV56 | This work |
| MB750 | Δ*sepH::apr* *attB_ΦBT1_*::P*_ftsZ_-ftsZ-ypet* | pSS5 integrated into the ΦBT1-attachment site of SV56 | This work |
| MB751 | WT *attB_ΦBT1_*::P*_sepH_-sepH-ypet attB_ΦC31_*::P*_ftsZ_-ftsZ-mcherry* | pMB196 integrated into the ΦBT1-attachment site and pSS29 integrated into the ΦC31-attachment site of *S. venezuelae* | This work |
| MB763 | WT *attB_ΦBT1_*::P*_sepH_-sepH-ypet* | pMB196 integrated into the ΦBT1-attachment site of *S. venezuelae* | This work |
| MB807 | WT *attB_ΦBT1_*::P*_sepH_-sepH-ypet; P_hupA_-hupA-mcherry* | pMB583 integrated into the ΦBT1-attachment site of *S. venezuelae* | This work |
| MB827 | Δ*sepH::apr* *attB_ΦBT1_*::P*_sepH_-sepH-CTD-y*pet | pMB592 integrated into the ΦBT1-attachment site of SV56 | This work |
| MB828 | Δ*sepH::apr* *attB_ΦBT1_*::P*_sepH_-sepH-NTD-y*pet | pMB591 integrated into the ΦBT1-attach-ment site of SV56 | This work |
| **Strain** | **Details** | **Source** | **Reference** |
| MB852 | Δ*sepH::apr* *attB_ΦBT1_*::*P_ermE_*- sepH-NTD-ypet* | pMB621 integrated into the ΦBT1-attachment site of SV56 | This work |
| MB851 | Δ*sepH::apr* *attB_ΦBT1_*::*_Z_- sepH-CTD-ypet* | pMB619 integrated into the ΦBT1-attachment site of SV56 | This work |
| MB856 | WT *attB_ΦBT1_*::*P_ermE_*- sepH-NTD-ypet* | pMB621 integrated into the ΦBT1-attachment site of WT | This work |
| MB858 | WT *attB_ΦBT1_*::*P_ermE_**- *sepH-ypet* | pMB618 integrated into the ΦBT1-attachment site of *S. venezuelae* | This work |
| MB859 | Δ*ftsZ::apr* *attB_ΦBT1_*::*P_ermE_**- *sepH-ypet* | pMB618 integrated into the ΦBT1-attachment site of DU669 | This work |
| MB918 | Δ*sepH::apr* *attB_ΦBT1_*::P*_sepH_-sepH-ypet* | pMB196 integrated into the ΦBT1-attachment site of SV56 | This work |
| MB938 | Δ*sepH::apr* *attB_ΦBT1_*:: *P_sepH_*-*-sepH-G79P* | pMB688 integrated into the ΦBT1-attachment site of SV56 | This work |
| SS380 | Δ*sepH*::*apr* *attB_ΦBT1_*::*P_sepH_*-*MSMEG*_5685-*mcherry* | pSS491 integrated into the ΦBT1-attachment site of SV56 | This work |
| *E. coli* | | | |
| ET12567(pUZ8002) | *F^–^ dam13::Tn9 dcm6 hsdM hsdR recF143::Tn10 galK2 galT22 ara-14 lacY1 xyl-5leuB6 thi-1 tonA31 rpsL hisG4 tsx-78 mtl-1 glnV44* | ET12567 containing helper plasmid pUZ8002 | Paget et al., 1999 |
| BW25113 | Δ(*araD*-*araB*)*567* Δ *lacZ4787*(::*rrnB-4*) *lacIp-4000*(*lacI^Q^*), l*-rpoS369*(*Am*) *rph-1* Δ(*rhaD*-*rhaB*)*568 hsdR514* | BW25113 containing λ RED recombination plasmid | Datsenko and Wanner, 2000 |
| DH5α | F^-^ *fhuA2* (*argF-lacZ*)U169 *phoA* *glnV44* 80 (*lacZ*)M15 *gyrA96*  *recA1* *relA1* *endA1* *thi-1* *hsdR17* | Cloning and membrane protein topology analysis | NEB |
| Rosetta (DE3) | F^-^ *ompT hsdS*_B_(r_B_^-^ m_B_^-^) *gal dcm* (DE3) pRARE (Cam^R^) | Host strain for protein overexpression | Merck |
| **Strain** | **Details** | **Source** | **Reference** |
| *S. cerevisiae* | | | |
| Y2HGold | *MATα, ura3-52, his3-200, ade2-101, trp1-901, leu2-3, 112, gal4Δ, met–, gal80Δ, MEL1, URA3::GAL1_UAS_ -GAL1_TATA_-lacZ* | Host strain for yeast-two hybrid analysis | Clontech |

**Table 2:** Plasmids used in this study.

| **Name** | **Description** | **Construction/References** |
| --- | --- | --- |
| pDONR207 | Gateway entry vector (Gent^R^) | Life Technologies |
| pGADCg | Yeast two-hybrid cloning vector, C-terminal GAL4 AD fusion (Amp^R^) | Clontech |
| pGADT7 | Yeast two-hybrid cloning vector, N-terminal GAL4 AD fusion (Amp^R^) | Clontech |
| pGBKCg | Yeast two-hybrid cloning vector, C-terminal GAL4 DNA-BD fusion (Kan^R^) | Clontech |
| pGBKT7 | Yeast two-hybrid cloning vector, N-terminal GAL4 DNA-BD fusion (Kan^R^) | Clontech |
| pET-21b | Plasmid for overexpression of proteins with N-terminal His_6_ fusion | Novagen |
| pTB145 | Plasmid for overexpression of His_6_-Upl1 protease (Amp^R^) | Bendezú et al., 2009 |
| pTB146 | Plasmid for overexpression of proteins with N-terminal His_6_-SUMO fusion (Amp^R^) | Bendezú et al., 2009 |
| pIJ10257 | Plasmid cloning vector for the conjugal transfer of DNA (under control of the *P_ermE_** constitutive promoter) from *E. coli* to *Streptomyces spp.* Integrates specifically at the ΦBT1 attachment site (Hyg^R^) | Hong et al., 2005 |
| pIJ773 | pBluescript KS (+) containing the apramycin resistance gene *apr* and *oriT* of plasmid RP4, flanked by FRT sites (Apr^R^). Used as template for the amplification of the *apr oriT* cassette for ‘Redirect’ PCR-targeting (Apr^R^) | Gust et al., 2003 |
| pIJ790 | Modified λ RED recombination plasmid *[oriR101] [repA101*(ts*)] araBp-gam-bet-exo* (Cam^R^) | Gust et al., 2003 |
| **Name** | **Description** | **Construction/References** |
| pMS82 | Plasmid cloning vector for the conjugal transfer of DNA from *E. coli* to *Streptomyces* spp. Integrates site specifically at the ΦBT1 attachment site (Hyg^R^) | Gregory et al., 2003 |
| pIJ10770 | Modified pIJ10750 lacking an intrinsic apramycin promoter upstream of the extended multiple cloning site (Hyg^R^) | Schlimpert et al., 2017 |
| pSS5 | pMS82 carrying *P_ftsZ_-ftsZ* (Hyg^R^) | Schlimpert et al., 2017 |
| pSS172 | pIJ10750 carrying *mcherry* (Hyg^R^) | Schlimpert et al., 2017 |
| pSS238 | pIJ10257 carrying *sepF* (Hyg^R^) | Schlimpert et al., 2017 |
| pSS245 | pIJ10257 carrying *sepF2* (Hyg^R^) | Schlimpert et al., 2017 |
| pSS250 | pGADT7 carrying *sepF2* (Amp^R^) | Schlimpert et al., 2017 |
| pSS250 | pGBKT7 carrying *sepF2* (Kan^R^) | Schlimpert et al., 2017 |
| pSS253 | pGBT9 carrying *ftsZ* (Amp^R^) | Schlimpert et al., 2017 |
| pSS254 | pGAD424 carrying *ftsZ* (Amp^R^) | Schlimpert et al., 2017 |
| pSS269 | pGADT7 carrying *dynA* (Amp^R^) | Schlimpert et al., 2017 |
| pSS270 | pGADT7 carrying *dynB* (Amp^R^) | Schlimpert et al., 2017 |
| pSS271 | pGADT7 carrying *sepF* (Amp^R^) | Schlimpert et al., 2017 |
| pSS272 | pGADT7 carrying *sepF3* (Amp^R^) | Schlimpert et al., 2017 |
| pSS273 | pGBKT7 carrying *dynB* (Kan^R^) | Schlimpert et al., 2017 |
| pSS274 | pGBKT7 carrying *dynA* (Kan^R^) | Schlimpert et al., 2017 |
| pSS275 | pGBKT7 carrying *sepF* (Kan^R^) | Schlimpert et al., 2017 |
| pSS276 | pGBKT7 carrying *sepF3* (Kan^R^) | Schlimpert et al., 2017 |
| pFRL10 | pGBKCg carrying *sepH* (Kan^R^) | Resulting from LR Gateway recombination between pFRL6-C and pGBKCg |
| pFRL11 | pGADCg carrying *sepH* (Amp^R^) | Resulting from LR Gateway recombination between pFRL6-C and pGADCg |
| pFRL12-N | pDONR207 carrying *sepH-NTD* (Gent^R^) | Amplification of *sepH*_1-186_ using primers fr8/fr12 cloned into pDONR207 using BP Gateway reaction |
| pFRL14-N | pDONR207 carrying *sepH-CTD* (Gent^R^) | Amplification of *sepH*_187-344_ using primers fr16/fr9 cloned into pDONR207 using BP Gateway reaction |
| pFRL15 | pGADT7 carrying *sepH-NTD* (Amp^R^) | Resulting from LR Gateway recombination between pFRL12-N and pGADT7 |
| pFRL23 | pGADT7 carrying *sepH-CTD* (Amp^R^) | Resulting from LR Gateway recombination between pFRL14-N and pGADT7 |
| pFRL39 | pTB146 carrying *sepH* (Amp^R^) | Amplification of *sepH* using primers fr34/fr35; amplification of pTB146 using primers 1219/1220. Assembly of both fragments using Gibson (NEB) |
| **Name** | **Description** | **Construction/References** |
| pFRL40 | pTB146 carrying *sepH-NTD* (Amp^R^) | Amplification of *sepH*_1-186_ using primers fr36/fr37; amplification of pTB146 using primers 1219/1220. Assembly of both fragments using Gibson (NEB) |
| pFRL41 | pTB146 carrying *sepH-CTD* (Amp^R^) | Amplification of *sepH*_187-344_ using primers fr38/fr39; amplification of pTB146 using primers 1219/1220. Assembly of both fragments using Gibson (NEB) |
| pFRL6-C | pDONR207 carrying *sepH* lacking stop codon (Gent^R^) | Amplification of *sepH* using primers fr8/fr10 cloned into pDONR207 using BP Gateway reaction |
| pFRL6-N | pDONR207 carrying *sepH* including stop codon (Gent^R^) | Amplification of *sepH* using primers fr8/fr9 cloned into pDONR207 using BP Gateway reaction |
| pFRL8 | pGBKT7 carrying *sepH* (Kan^R^) | Resulting from LR Gateway recombination between pFRL6-N and pGBKT7 |
| pFRL9 | pGADT7 carrying *sepH* (Amp^R^) | Resulting from LR Gateway recombination between pFRL6-N and pGADT7 |
| pJUK10 | pGBKT7 carrying *sepH_ms_* (Kan^R^) | Recombinant plasmid resulting from LR Gateway reaction between pJUK7 and pGBKT7 |
| pJUK12 | pGADT7 carrying *sepH_ms_* (Amp^R^) | Recombinant plasmid resulting from LR Gateway reaction between pJUK7 and pGADT7 |
| pJUK15 | pGBKCg carrying *sepF_ms_* (Kan^R^) | Recombinant plasmid resulting from LR Gateway reaction between pJUK8 and pGBKCg |
| pJUK17 | pGADCg carrying *sepF_ms_* (Amp^R^) | Recombinant plasmid resulting from LR Gateway reaction between pJUK8 and pGADCg |
| pJUK19 | pGBKCg carrying *ftsZ_ms_* (Kan^R^) | Recombinant plasmid resulting from LR Gateway reaction between pJUK9 and pGBKCg |
| pJUK21 | pGADCg carrying *ftsZ_ms_* (Amp^R^) | Recombinant plasmid resulting from LR Gateway reaction between pJUK9 and pGADCg |
| pJUK7 | pDONR207 carrying *sepH_ms_* (Gent^R^) | Amplification of *sepH_ms_* using primers JS25/JS26 cloned into pDONR207 using BP Gateway reaction |
| pJUK8 | pDONR207 carrying *sepF_ms_* (Gent^R^) | Amplification of *sepF_ms_* using primers JS27/JS28 cloned into pDONR207 using BP Gateway reaction |
| pJUK9 | pDONR207 carrying *ftsZ_ms_* (Gent^R^) | Amplification of *ftsZ_ms_* using primers JS29/JS30 cloned into pDONR207 using BP Gateway reaction |
| **Name** | **Description** | **Construction/References** |
| pMB196 | pMS82 carrying *P_sepH_-sepH-ypet* (Hyg^R^) | Two-step amplification using mb177/mb398 and mb399/mb261 in the first step. Templates from first step amplified using mb177 and mb261 to generate *P_sepH_-sepH-[SGGGG]*-*ypet* fragment, cloned via HindIII/KpnI into pMS82 |
| pMB557 | pIJ10770 carrying *P_seH_-sepH* (Hyg^R^) | Amplification of *P_sepH_-sepH* using mb177/mb213 and cloned into pIJ10770 via HindIII/KpnI |
| pMB583 | pSS305 carrying *P_sepH_-sepH-ypet* (Hyg^R^) | *psepH-sepH-ypet* was amplified by mb1019/mb1020 and cloned via EcoRV into pSS305 |
| pMB587 | pMS82 carrying *P_sepH_-sepH-CTD-y*pet (Hyg^R^) | Two-step amplification using pMB196 as a template and primer mb1032/mb1036 and mb1037/mb1035 in the first step. Templates from the first step were annealed and amplified using mb1032 and mb1035 to generate the *P_sepH_-sepH-CTD-y*pet fragment, cloned via HindIII/KpnI into pMS82 |
| pMB588 | pMS82 carrying *P_sepH_-sepH-NTD-ypet* (Hyg^R^) | Two-step amplification using pMB196 as a template and primer mb1032/mb1033 and mb1034/1035 in the first step. Templates from the first step were annealed and amplified using mb1032 and mb1035 to generate *P_sepH_-sepH-NTD-ypet* fragment, cloned via HindIII/KpnI into pMS82 |
| pMB591 | pIJ10770 carrying *P_sepH_-sepH-NTD-ypet* (Hyg^R^) | *P_sepH_-sepH-NTD-ypet* was sub-cloned from pMB588 via HindIII/KpnI |
| pMB592 | pIJ10770 carrying *P_sepH_-sepH-CTD-ypet* (Hyg^R^) | *P_sepH_-sepH-CTD-ypet* was subcloned from pMB587 via HindIII/KpnI |
| pMB618 | pIJ10257 carrying *P_sepH_-sepH-ypet* (Hyg^R^) | Amplification of *sepH-ypet* using pMB196 as a template and primer mb1068/mb1069 and cloned into pIJ10257 using NdeI/HindIII |
| pMB619 | pIJ10257 carrying *sepH-CTD-ypet* (HygR) | Amplification of *P_sepH_-sepH-CTD-ypet* using pMB587 as a template and primer mb1070/mb1069 – cloned via NdeI/HindIII into pIJ10257 |
| pMB621 | pIJ10257 carrying *sepH-NTD-ypet* (Hyg^R^) | Amplification of *P_sepH_-sepH-NTD-ypet* using pMB588 as a template and primer mb1068/mb1069 – cloned via NdeI/HindIII into pIJ10257 |
| pMB674 | pGADT7 carrying *sepH-G79P* (Amp^R^) | *Pfu-UltraII*-Quikchange of pFRL9 using mb1122/mb1123 |
| pMB688 | pIJ10770 carrying *psepH-sepH-G79P* (Hyg^R^) | *Pfu-UltraII*-Quikchange of pMB557 using mb1122/mb1123 |
| **Name** | **Description** | **Construction/References** |
| pSS29 | pKF351 carrying *P_ftsZ_-ftsZ-mcherry* | *ypet* was removed from pKF351 by restriction digestion with NdeI/BglII and replaced by *mcherry* |
| pSS287 | pTB146 carrying *ftsZ* (Amp^R^) | Insert and recipient vector were PCR amplified with oligo 452/453 and 451/454, respectively, followed by Gibson assembly |
| pSS305 | pSS172 carrying *hupA* (Hyg^R^) | Insert was PCR amplified with oligo ss103/ss104, cut with HindIII/AvrII followed by ligation into pSS172 cut with HindIII/AvrII |
| pSS472 | pIJ10257 carrying *msmeg_5685-mcherry* (Hyg^R^) | Codon-optimised *msmeg_5685* was PCR amplified with oligo ss1018/ss1020 and partially cut with NdeI/XhoI followed by ligation into pSS426 cut with NdeI-XhoI |
| pSS491 | pIJ10257-derivative carrying *msmeg_5685-mcherry* (Hyg^R^) | *sepH* promoter region was PCR amplified with oligo ss1055/ss1056, cut with KpnI/NdeI. This fragment was used to replace the *P_ermE_** fragment in pSS472 |
| pSS560 | pTB146 carrying *ftsZ_Ms_* (Carb^R^) | Amplification of *MSMEG_4222* using oligo ss1221/ss1222 and assembled with pTB146 which was amplified with primer ss1219/ss1220 |
| pSS561 | pET21b carrying *sepH_Ms_* (Carb^R^) | Amplification of *MSMEG_5685* using oligo ss1225/ss1226 and insertion between the NdeI/HindIII site of pET21b via restriction cloning |

**Table 3: Oligonucleotides used in this work.**

| **Primer** | **Sequence** |
| --- | --- |
| ss103 | attaAAGCTTgccgctcggacttcccgaaccag |
| ss104 | ttaattCCTAGGcttgcccttggcggcttccttgag |
| ss451 | ACCACCAATCTGTTCTCTGTGAG |
| ss452 | GAGAACAGATTGGTGGTATGGCAGCACCGCAGAAC |
| ss453 | GGAGCTCTGCTCTTCTTCACTTCAGGAAGTCCGGG |
| ss454 | AGAAGAGCAGAGCTCCGTCGAC |
| ss1018 | AATTAATTCATATGCGGGAGCTGCGCGTGGTGG |
| ss1020 | ATATACTCGAGCCGCTGGGTGCCCGACGACC |
| ss1055 | ATCGGGGTACCCCCGCTACCTGCACATCGACGG |
| ss1056 | AATTAATTCATATGTCACACGGTAGAGGGGTTCGCG |
| ss1219 | CTCCGTCGACAAGCTTGCGG |
| ss1220 | ACCACCAATCTGTTCTCTGTGAGCC |
| ss1221 | GAACAGATTGGTGGTATGACCCCCCCGCATAACTACCTCG |
| ss1222 | CCGGGCTCGAGTGCGTCAGTGCCGCATGAAGGGCGG |
| ss1225 | attaattcatatgcgagaactcagggtcgtcggac |
| ss1226 | attaAAGCTTgcgctgcgtacccgacgagc |
| fr10 | GGGGACCACTTTGTACAAGAAAGCTGGGTGGTCCTGCTTCTTCCGCCGCGTG |
| **Primer** | **Sequence** |
| fr12 | GGGGACCACTTTGTACAAGAAAGCTGGGTGTCAGATCAGGGCCCTGGCCTC |
| fr16 | GGGGACAAGTTTGTACAAAAAAGCAGGCTTCATGGGCGAGACGGACGACACG |
| fr34 | ACAGAGAACAGATTGTGTGTATGCCCGAACTGCGTGTC |
| fr35 | CCGCAAGCTTGTCGACGGAGTCAGTCCTGCTTCTTCCG |
| fr36 | ACAGAGAACAGATTGGTGGTATGCCCGAACTGCGTGTC |
| fr37 | CCGCAAGCTTGTCGACGGAGTCAGCCGATCAGGGCCCT |
| fr38 | ACAGAGAACAGATTGGTGGTGGCGAGACGGACGACACG |
| fr39 | CCGCAAGCTTGTCGACGGAGTCAGTCCTGCTTCTTCCGC |
| fr8 | GGGGACAAGTTTGTACAAAAAAGCAGGCTTCATGCCCGAACTGCGTGTCGTGG |
| fr9 | GGGGACCACTTTGTACAAGAAAGCTGGGTGTCAGTCCTGCTTCTTCCGCCG |
| JS25 | GGGGACAAGTTTGTACAAAAAAGCAGGCTTCATGCGAGAACTCAGGGTCGTCG |
| JS26 | GGGGACCACTTTGTACAAGAAAGCTGGGTGTCAGCGCTGCGTACCCGA |
| JS27 | GGGGACAAGTTTGTACAAAAAAGCAGGCTTCATGAGCACACTGCATAAGGT |
| JS28 | GGGGACCACTTTGTACAAGAAAGCTGGGTGACGGTAGGAGTAGAAGCCCG |
| JS29 | GGGGACAAGTTTGTACAAAAAAGCAGGCTTCATGACCCCCCCGCATAACT |
| JS30 | GGGGACCACTTTGTACAAGAAAGCTGGGTGGTGCCGCATGAAGGGCGG |
| mb118 | CACGTGACGTCGGCAGGCACCACCCGGGAGGTCCCCATGATTCCGGGGATCCGTCGACC |
| mb119 | AGCCGCGGAACCGGCGGACCGCCACGGCTCCTGCCGTCATGTAGGCTGGAGCTGCTTC |
| mb144 | GGCAGGCCACACTCGACT |
| mb145 | ACCGGACCCGCAGTAGTC |
| mb177 | GGCGAAGCTTCCCCTGCTCCAGTCCCTG |
| mb213 | GGGGTACCGACCGCCACGGCTCCTG |
| mb261 | GGGGTACCTCACTTGTACAGCTCGTTCATG |
| mb398 | CTTGGAGACGCCGCCGCCGCCACTGTCCTGCTTCTTCCGCCG |
| mb399 | AAGCAGGACAGTGGCGGCGGCGGCGTCTCCAAGGGCGAGGAGC |
| mb1019 | GGGCGGATATCCCCCTGCTCCAGTCCCTG |
| mb1020 | GGGCGGATATCTCACTTGTACAGCTCGTTCATGC |
| mb1032 | GGCGAAGCTTCCCCTGCTCCAGTCCCTG |
| mb1033 | GCCGCCACTGATCAGGGCCCTGGCCTC |
| mb1034 | GCCCTGATCAGTGGCGGCGGCGGCGTC |
| mb1035 | GGGGTACCTCACTTGTACAGCTCGTTCATGCCC |
| mb1036 | CGTCTCGCCCATGGGGACCTCCCGGGT |
| mb1037 | GTCCCCATGGGCGAGACGGACGACACG |
| mb1068 | GGGAATTCCATATGCCCGAACTGCGTGTCGTGG |
| mb1069 | CCCAAGCTTTCACTTGTACAGCTCGTTCATGCCC |
| mb1070 | GGGAATTCCATATGGGCGAGACGGACGACACGA |
| mb1122 | GCCCAGATGGCCCCGATCCCCGTCGAC |
| mb1123 | GTCGACGGGGATCGGGGCCATCTGGGC |
| mb1124 | GGCATCGCCGAGCTCCGCG |
| mb1125 | GCGGTCGTCGCCGCGGGC |
| mb1126 | ATGAGCCATATTCAACGGGAAACGTC |
| mb1127 | CCATGCATCATCAGGAGTACGGATA |
| mb1129 | CCTTCGATCGTCCCTGGTCGGAGC |
| **Primer** | **Sequence** |
| mb1133 | GGCCTGGCGCTGGGGCTT |
| mb1136 | GCCCTTCGCGTCACGGCG |
| mb1138 | GCTGCTCGTCCCCGCGCA |
| mb1139 | GCGGCTGACACCGAGTGC |
| mb1140 | GATCATCGCGAGCCGCGGCAA |

**Supplementary References**

Bendezú FO, Hale CA, Bernhardt TG, de Boer PAJ. 2009. RodZ (YfgA) is required for proper assembly of the MreB actin cytoskeleton and cell shape in *E. coli*. *EMBO J* **28**:193–204. doi:10.1038/emboj.2008.264

Datsenko KA, Wanner BL. 2000. One-step inactivation of chromosomal genes in *Escherichia coli* K-12 using PCR products. *PNAS* **97**:6640–6645. doi:10.1073/pnas.120163297

Gregory MA, Till R, Smith MCM. 2003. Integration site for *Streptomyces* phage phiBT1 and development of site-specific integrating vectors. *J Bacteriol* **185**:5320–5323. doi:10.1128/jb.185.17.5320-5323.2003

Gust B, Challis GL, Fowler K, Kieser T, Chater KF. 2003. PCR-targeted *Streptomyces* gene replacement identifies a protein domain needed for biosynthesis of the sesquiterpene soil odor geosmin. *PNAS* **100**:1541–1546. doi:10.1073/pnas.0337542100

Hong H-J, Hutchings MI, Hill LM, Buttner MJ. 2005. The role of the novel Fem protein VanK in vancomycin resistance in *Streptomyces coelicolor*. *J Biol Chem* **280**:13055–13061. doi:10.1074/jbc.M413801200

Paget MSB, Chamberlin L, Atrih A, Foster SJ, Buttner MJ. 1999. Evidence that the extracytoplasmic function sigma factor σ^E^ is required for normal cell wall structure in *Streptomyces coelicolor* A3(2). *J Bacteriol* **181**:204–211.

Santos-Beneit F, Roberts DM, Cantlay S, McCormick JR, Errington J. 2017. A mechanism for FtsZ-independent proliferation in *Streptomyces*. *Nat Commun* **8**:1378. doi:10.1038/s41467-017-01596-z

Schlimpert S, Wasserstrom S, Chandra G, Bibb MJ, Findlay KC, Flärdh K, Buttner MJ. 2017. Two dynamin-like proteins stabilize FtsZ rings during *Streptomyces* sporulation. *Proc Natl Acad Sci USA* **114**:E6176–E6183. doi:10.1073/pnas.1704612114
