# Supplementary material for: A conserved cell division protein directly regulates FtsZ dynamics in filamentous and unicellular actinobacteria
Source: eLife. 2021 Mar 17;10:e63387. doi: 10.7554/eLife.63387 (PMC7968930; doi:10.7554/eLife.63387)
Supplement: Transparent reporting form [file elife-63387-transrepform.docx]

***eLife’s* transparent reporting form**

We encourage authors to provide detailed information *within their submission* to facilitate the interpretation and replication of experiments. Authors can upload supporting documentation to indicate the use of appropriate reporting guidelines for health-related research (see [EQUATOR Network](http://www.equator-network.org/%20)), life science research (see the [BioSharing Information Resource](https://biosharing.org/)), or the [ARRIVE guidelines](http://www.plosbiology.org/article/info:doi/10.1371/journal.pbio.1000412) for reporting work involving animal research. Where applicable, authors should refer to any relevant reporting standards documents in this form.

If you have any questions, please consult our Journal Policies and/or contact us: [editorial@elifesciences.org](mailto:editorial@elifesciences.org).

**Sample-size estimation**

- You should state whether an appropriate sample size was computed when the study was being designed
- You should state the statistical method of sample size computation and any required assumptions
- If no explicit power analysis was used, you should describe how you decided what sample (replicate) size (number) to use

Please outline where this information can be found within the submission (e.g., sections or figure legends), or explain why this information doesn’t apply to your submission:

No power analysis was employed to prepare statistical samples. For assays and analyses used in this study, technical and biological replicates are the primary concern.

Figure legends contain information on sample size and number of replicates. For instance, in the legend of Figure 7B we state “the mean ± SD spore length for each strain are denoted below. 350 spores per replicate (n=3) per strain were measured.”

**Replicates**

- You should report how often each experiment was performed
- You should include a definition of biological versus technical replication
- The data obtained should be provided and sufficient information should be provided to indicate the number of independent biological and/or technical replicates
- If you encountered any outliers, you should describe how these were handled
- Criteria for exclusion/inclusion of data should be clearly stated
- High-throughput sequence data should be uploaded before submission, with a private link for reviewers provided (these are available from both GEO and ArrayExpress)

Please outline where this information can be found within the submission (e.g., sections or figure legends), or explain why this information doesn’t apply to your submission:

Figure legends contain information on sample size and number of replicates. For instance, in the legend of Figure 7B we state “For comparison spore chains of the wildtype and the Δ*sepH* mutant (SV56) are shown and the mean ± SD spore length for each strain are denoted below. 350 spores per biological replicate (n=3) and strain were measured.”

The SepH ChIP-seq data has been deposited at the MIAME-compliant ArrayExpress database under accession number E-MTAB-9064.

**Statistical reporting**

- Statistical analysis methods should be described and justified
- Raw data should be presented in figures whenever informative to do so (typically when N per group is less than 10)
- For each experiment, you should identify the statistical tests used, exact values of N, definitions of center, methods of multiple test correction, and dispersion and precision measures (e.g., mean, median, SD, SEM, confidence intervals; and, for the major substantive results, a measure of effect size (e.g., Pearson's r, Cohen's d)
- Report exact p-values wherever possible alongside the summary statistics and 95% confidence intervals. These should be reported for all key questions and not only when the p-value is less than 0.05.

Please outline where this information can be found within the submission (e.g., sections or figure legends), or explain why this information doesn’t apply to your submission:

We provide P-values only in Figure 1-supplement figure 2. Details about sample size, number of replicates and statistical analysis are provided in the figure legend.

In case of biological replicates, the SD, SEM or CIs are shown and explained in the figure legends.

(For large datasets, or papers with a very large number of statistical tests, you may upload a single table file with tests, Ns, etc., with reference to sections in the manuscript.)

**Group allocation**

- Indicate how samples were allocated into experimental groups (in the case of clinical studies, please specify allocation to treatment method); if randomization was used, please also state if restricted randomization was applied
- Indicate if masking was used during group allocation, data collection and/or data analysis

Please outline where this information can be found within the submission (e.g., sections or figure legends), or explain why this information doesn’t apply to your submission:

No special method for group allocation was used. For each replicate, experimental and control groups were imaged or measured, processed and analyzed together. A detailed description of the processing and analysis is reported in the figure legends and/or Materials and Methods section.

**Additional data files (“source data”)**

- We encourage you to upload relevant additional data files, such as numerical data that are represented as a graph in a figure, or as a summary table
- Where provided, these should be in the most useful format, and they can be uploaded as “Source data” files linked to a main figure or table
- Include model definition files including the full list of parameters used
- Include code used for data analysis (e.g., R, MatLab)
- Avoid stating that data files are “available upon request”

Please indicate the figures or tables for which source data files have been provided:

No large data sets were used in this manuscript.

Where considered appropriate and as indicated in the figure legends, source data used to generate figures has been uploaded to eLife, including:

- image stack used to generate kymographs shown in Figure 2

- tree file and alignment files used to generate Figure 7A and Figure 7-supplement 1.

- Excel spread sheets containing data used in e.g. spore size measurements, FtsZ filament measurements, calculation of protein sedimentation efficiency or predicted protein multimerization states.
